# Identifying the serious clinical outcomes of adverse reactions to drugs by a multi-task deep learning framework

**DOI:** 10.1038/s42003-023-05243-w

**Published:** 2023-08-24

**Authors:** Haochen Zhao, Peng Ni, Qichang Zhao, Xiao Liang, Di Ai, Shannon Erhardt, Jun Wang, Yaohang Li, Jianxin Wang

**Affiliations:** 1 School of Computer Science and Engineering, Central South University, Changsha, 410083 China; 2 Hunan Provincial Key Lab on Bioinformatics, Central South University, Changsha, 410083 China; 3 Xiangjiang Laboratory, Changsha, 410205 China; 4 Department of Pathology and Laboratory Medicine, McGovern Medical School, The University of Texas Health Science Center at Houston, Houston, TX 77030 USA; 5 Department of Pediatrics, McGovern Medical School, The University of Texas Health Science Center at Houston, Houston, TX 77030 USA; 6 Department of Computer Science, Old Dominion University, Norfolk, VA 23529-0001 USA

**Keywords:** Computational models, Outcomes research

## Abstract

Adverse Drug Reactions (ADRs) have a direct impact on human health. As continuous pharmacovigilance and drug monitoring prove to be costly and time-consuming, computational methods have emerged as promising alternatives. However, most existing computational methods primarily focus on predicting whether or not the drug is associated with an adverse reaction and do not consider the core issue of drug benefit-risk assessment—whether the treatment outcome is serious when adverse drug reactions occur. To this end, we categorize serious clinical outcomes caused by adverse reactions to drugs into seven distinct classes and present a deep learning framework, so-called GCAP, for predicting the seriousness of clinical outcomes of adverse reactions to drugs. GCAP has two tasks: one is to predict whether adverse reactions to drugs cause serious clinical outcomes, and the other is to infer the corresponding classes of serious clinical outcomes. Experimental results demonstrate that our method is a powerful and robust framework with high extendibility. GCAP can serve as a useful tool to successfully address the challenge of predicting the seriousness of clinical outcomes stemming from adverse reactions to drugs.

## Abstract (web summary)

A deep-learning framework predicts whether adverse drug interactions (ADRs) lead to serious clinical outcomes and identifies the corresponding classes based on the drug structures, ADR semantic features, and known drug-ADR interactions.

## Introduction

When a drug receives market approval, it must not only prove its effectiveness for patients but also demonstrate that its expected benefits outweigh its potential risks^1,2^—essentially, it must undergo a risk-benefit assessment. A key concern in the risk-benefit assessment of drugs is to determine the possible treatment outcomes of patients if the drug produces adverse reactions^3,4^. Adverse Drug Reactions (ADRs) refer to an appreciably harmful or unpleasant reaction resulting from an intervention related to the use of a medicinal product. In reality, no drug is free of adverse reaction risks; some can be life-threatening or even directly fatal^5,6^. ADRs negatively impact people worldwide, and the expenses related to hospitalization and surgery due to adverse reactions can sometimes surpass the cost of the drug treatment itself^7,8^. Statistical data reveal that tens of billions of dollars are spent annually in the United States to address the myriad public health issues arising from ADRs^9^. Therefore, early detection and assessment of the seriousness of clinical outcomes associated with adverse reactions to new drugs are crucial for safeguarding patient health and assessing assess the risk-benefit of drugs^10,11^.

Unfortunately, many ADRs with serious outcomes are not observed during clinical trials^12^. For example, fenfluramine, an appetite suppressant, was approved in the U.S. in 1973^13^. However, hundreds of drug users in the U.S. have been found to have developed potentially fatal cardiovascular diseases. Due to these cases, the U.S. Food and Drug Administration (FDA) forcibly withdraw fenfluramine from the market in 1997. Moreover, some drug adverse reactions are often coincide with varying clinical outcomes. For example, diarrhea is a common adverse reactions to some drugs^14^. Mild diarrhea usually does not require specific treatment and resolves gradually over time. However, serious diarrhea can persist for days, causing dehydration and salt depletion, potentially leading to death^15^. Therefore, if the clinical outcomes of adverse reactions to drugs can be predicted and assessed before it is marketed, the risk to patients and the failure to drug development can be reduced before the drugs are released to the market^16^.

Traditional ADR identification relies on collecting adverse drug events (ADEs) during preclinical research and clinical trials^17^. The whole process is time-consuming and the number of patient samples is limited^18^. Consequently, the detection of ADRs depends heavily on post-marketing surveillance, which involves systematically detecting and evaluating drug effects after they are marketed^19^. Computational methods can provide crucial guidance for trials. In recent years, numerous computational methods have been developed to assign potential ADRs based on heterogeneous drug databases^20–24^. For example, Huynh et al. ^25^ proposed two deep learning models, named convolutional recurrent neural networks and convolutional neural networks with attention (CNNA), for ADR prediction. These models were trained and evaluated using a Twitter dataset containing informal language and an ADE dataset constructed from MEDLINE case reports. Yu et al. ^26^ proposed a hybrid embedding graph neural network model, called idse-HE, to identify potential drug side effects. The model integrates a graph embedding module and a node embedding module, which can fuse drug chemical structure information, drug substructure sequence information, and drug network topology information to extract effective features. The drug-side effect interaction matrix is reconstructed and potential side effects of drugs are predicted by considering the final representation of drugs and side effects as two implicit factors. However, most existing computational methods on ADR prediction focus on the presence or absence of an drug–ADR interactions, failing to answer an important question raised by doctors and pharmaceutical companies: how can the clinical outcomes of adverse drug reactions be determined? ADRs with serious treatment outcomes may delay patient treatment, impact the quality of life, and increase treatment costs. Assessment of the seriousness of clinical outcomes of ADRs is essential for prioritizing patient care, enabling prompt medical intervention, guiding treatment adjustments, evaluating risk-benefit profiles, and informing regulatory decisions. By understanding the seriousness of the clinical outcomes of ADRs, healthcare professionals can take appropriate actions to protect patient health and ensure the safe and effective use of medications. Simultaneously, drugs that cause serious treatment outcomes may also face market withdrawal risks.

Therefore, there is a manifest need for addressing the following challenges: (1) accurately and efficiently identifying adverse reactions to drugs that cause serious clinical outcomes, taking account of information from both drugs and ADRs, which can reduce drug harm to patients; (2) ensuring generalization capability across large datasets; and (3) predicting the corresponding classes of serious clinical outcomes resulting from adverse reactions to drugs, which can reduce the risk of drug withdrawal from the market, protecting the patients, and minimize losses for pharmaceutical companies.

In this paper, we initially determine the drug–ADR interactions that cause serious clinical outcomes and quantify them into seven seriousness classes, namely: DEath (DE), Life-Threatening (LT), HOspitalization-Initial or prolonged (HO), DiSability (DS), Congenital Anomaly (CA), Required intervention to prevent permanent impairment/damage (RI), and OTher (OT), as documented in the FDA Adverse Event Reporting System (FAERS)^27^. Subsequently, we present GCAP, an end-to-end multi-task deep-learning framework for simultaneously predicting whether the drug–ADR interactions cause serious clinical outcomes and identifying the corresponding classes of serious clinical outcomes. This is achieved by considering the drug structures, ADR semantic features, and known drug–ADR interactions that result in serious clinical outcomes.

More specifically, we first collect the Simplified Molecular Input Line Entry System (SMILES) sequences of drugs and semantic descriptors of ADRs from PubChem^28^ and ADReCS^29^ databases respectively. Then, for a drug, we construct a drug molecule graph and encode the SMILES sequence as a dense numeric matrix. We also create a directed acyclic graph (DAG) for each ADR, encompassing all the semantic descriptors related to that ADR. Thirdly, to learn a richer and more accurate representation, we feed drug molecule graphs into a Graph Neural Network (GNN) module and SMILES sequence numerical matrices into a Convolutional Neural Network (CNN) module respectively. The feature vectors of ADRs are learned from the DAGs by calculating the related semantic descriptors of the ADRs. Finally, we stack the drug representations, ADR representation, and known interaction representations between drugs and ADRs, and input them into the Fusion module with a multi-head attention mechanism. Experimental results show that GCAP well on benchmark and independent datasets. Ablation experiments and visual analyses further illustrate the utility of our method for predicting the serious clinical outcomes of adverse reactions to drugs. In addition, we investigate the extended applications of the seriousness of clinical outcomes of drug–ADR interactions in three drug-related tasks, including a drug–drug interaction prediction, a drug response prediction, and a drug side-effect frequency prediction. The introduction of serious clinical outcomes of adverse reactions to drugs improves several state-of-the-art methods. Collectively, these results indicate that GCAP is a powerful and robust framework for identifying the serious clinical outcomes of adverse drug reactions, offering a valuable tool for researchers, clinicians, and the pharmaceutical industry, enabling more informed decision-making in drug development and clinical practice.

## Results

### Overview of GCAP

**Fig. 1:**
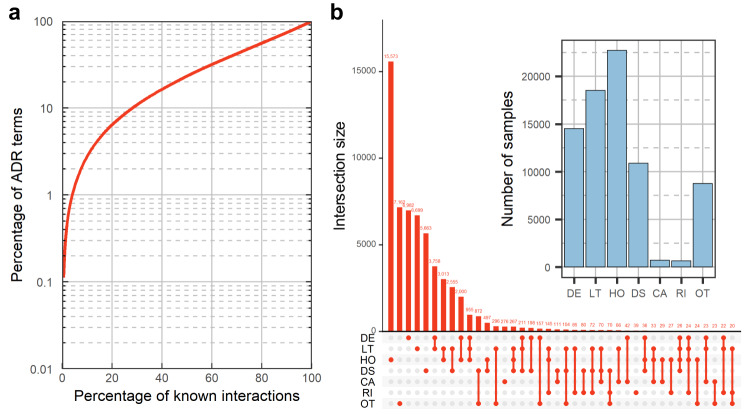
Distribution of known drug–ADR interactions and their corresponding clinical outcomes in our benchmark dataset. **a** Long-tailed distribution of ADRs. ADRs in the vertical axis are ordered in decreasing order of popularity, i.e., the number of drugs in which a particular ADR appears. **b** The upset plot shows the detailed number of drug–ADR interactions in each class group of serious clinical outcomes. In the upset plot, the y-axis represents the number of drug–ADR interactions that cause serious clinical outcomes, while the x-axis represents the components of each group. The bar chart shows the distribution of the drug–ADR interactions in each serious clinical outcomes’ class.

We use FAERS to obtain the clinical outcomes of adverse reactions to drugs. Following common practice in clinical trials and the definition of FAERS, we employ seven classes—OT, RI, CA, DS, HO, LT, and DE—to quantify the serious clinical outcomes caused by drug–ADR interactions. We construct a benchmark dataset consisting of 141,752 drug–ADR interactions, of which 58,429 drug–ADR interactions that cause serious clinical outcomes have classification labels for serious clinical outcome’s classes. Figure 1a shows the distribution of known drug–ADR interactions in the benchmark dataset, indicating the adverse reactions to drugs follow a long-tailed distribution (about 40% of the ADRs are responsible for 80% of the interactions). Figure 1b illustrates the distribution of the number of drug–ADR interactions in each serious clinical outcomes’ class in the benchmark dataset, indicating that seriousness from clinical trials are biased towards OT, DS, HO, LT and DE.

**Fig. 2:**
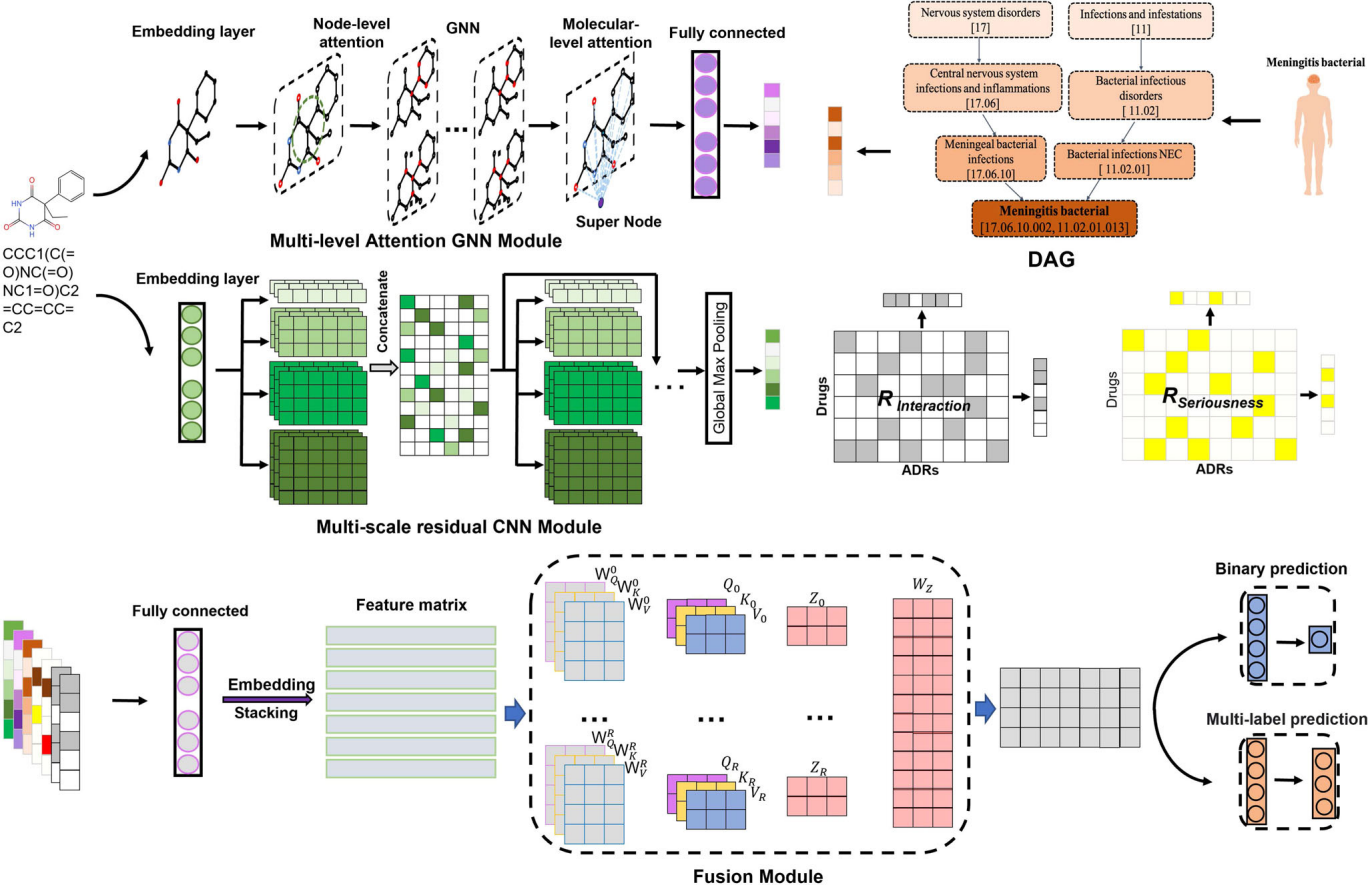
The network architecture of GCAP. Given a SMILES sequence of a drug and the semantic descriptors of an ADR, a drug molecule graph, a drug SMILES encoding matrix, and a semantic feature vector of the ADR can be constructed. The multi-level graph attention module and multi-scale residual CNN module are then used to extract representations from the drug molecule graph and SMILES encoding matrix, respectively. The semantic feature vector of an ADR is calculated based on all associated descriptors, and a fully connected layer is used to extract the representation from the semantic feature vector. Simultaneously, multiple fully-connected layers generate drug and ADR representations from the clinical outcomes of known drug–ADR interactions. All representations are then stacked and fed into a multi-head self-attention module to fuse multiple representations into a joint vector for downstream predictions. By setting different downstream classifiers for each task, GCAP can accurately predict potential drug–ADR interactions that cause serious clinical outcomes and identify the corresponding classes of serious clinical outcomes.

Figure 2 shows the overall network architecture of GCAP for predicting the serious clinical outcomes of adverse reactions to drugs. GCAP can be segmented into three main stages: representation learning of drugs and ADRs, representation fusion of drug–ADR interactions, and seriousness prediction of clinical outcomes for adverse reactions to drugs. Given the SMILES sequences and semantic descriptors of the input drugs and ADRs, GCAP exploits a multi-level attention GNN module, a multi-scale residual CNN module and the semantic DAGs to learn the representations of drugs and ADRs separately. The GNN module contains an atom-level and molecular-level attention mechanism to efficiently capture the hidden critical linkage among any nodes while respecting the intrinsic molecule topological structure of the drug. The CNN module contains convolutional filters of multiple scales to perform representation learning on SMILES sequences of drugs, and reduce the problem of vanishing or exploding gradients by adding residual paths. The DAGs describe the relationships among semantic descriptors of ADRs. We calculate the semantic descriptors in a DAG to get the feature vectors of ADRs.

In addition, we assemble two *n* $\times$ *m* matrices *R*_*Interaction*_ and *R*_*seriousness*_, which respectively containing all known drug–ADR interactions and the drug–ADR interactions that cause serious clinical outcomes for *n*  =  1073 unique drugs and *m*  =  893 unique ADRs in the benchmark dataset (Supplementary Data 1 and 2). The remaining entries in *R*_*Interaction*_ and *R*_*seriousness*_ are filled with zeros, indicating no interactions. Based on common assumptions that similar drugs tend to exhibit similar interaction and non-interaction patterns with ADRs, and vice versa, we use four connected layers to extract higher-order representations from the row and column of matrices *R*_*Interaction*_ and *R*_*seriousness*_ for drugs and ADRs respectively (each row of *R*_*Interaction*_ and *R*_*seriousness*_ is a drug representation and each column of *R*_*Interaction*_ and *R*_*seriousness*_ is an ADR representation). Next, we employ fully connected layers to unify the dimensions of seven representations and design a multi-head attention architecture to fuse the representations. Finally, we expand the fused vectors and set up two separate Multi-Layer Perception (MLP) modules for each task to obtain prediction results. The model’s input consists of the SMILES sequence of a drug, the semantic feature vector of an ADR, and the known clinical outcomes related to the drug and ADR.

The task of predicting potential drug–ADR interactions that cause serious clinical outcomes can be described as a binary classification problem, in which the label = 1 or 0 indicates the interaction between the input drug and ADR causes a serious or non-serious clinical outcome, respectively. GCAP can output a value ranging from 0 to 1, indicating the probability of a serious clinical outcome for the interaction between the input drug and the ADR. The task of determing the serious clinical outcomes’ classes for drug–ADR interactions can be described as a multi-label classification problem. In this case, we use a 7-dimensional vector to indicate the classes of serious clinical outcomes caused by drug–ADR interactions, where each real number in the vector indicates the probability of a drug–ADR interaction has the corresponding serious clinical outcome class. The value in predictive vectors for this task can also be denoted by a real value between 0 and 1.

### Identifying the seriousness of clinical outcomes of adverse reactions to drugs

**Fig. 3:**
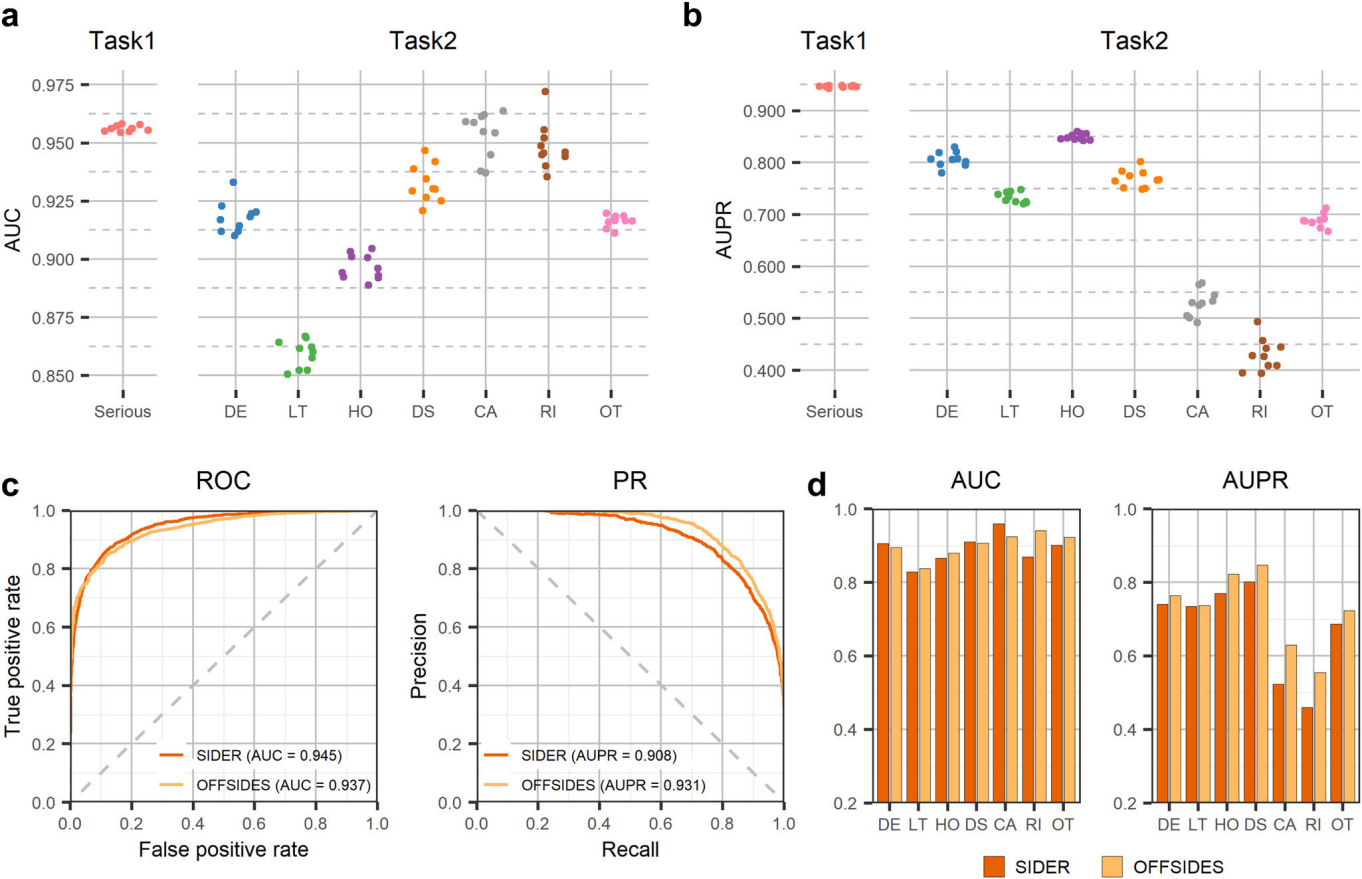
Evaluation of our method predictive performance on identifying the seriousness of clinical outcomes of adverse reactions to drugs. **a** and **b** show the distributions of AUCs and AUPRs on the task of identifying whether the adverse reactions to drugs cause serious clinical outcomes and the task of predicting the corresponding classes of serious clinical outcomes caused by seirous adverse reactions to drugs, respectively. The results are obtained by tenfold cross-validations based on the benchmark dataset. **c** depicts the Receiver Operating Characteristic (ROC) and Precision-Recall (PR) curves on the task of identifying whether the adverse reactions to drugs cause serious clinical outcomes based on two independent datasets, respectively. **d** shows the distributions of AUCs and AUPRs in the task of predicting the corresponding classes of serious clinical outcomes caused by serious adverse reactions to drugs.

To evaluate the performance of GCAP, ten times repeated 10-Fold Cross-Validation (10 × 10-Fold CV) and independent tests are conducted based on the benchmark dataset and two independent test sets, respectively (see Methods). The Area Under the receiver operating Characteristics curve (AUC) and the Area Under the Precision-Recall curve (AUPR) are utilized to evaluate the performance of GCAP. We start from analyzing the predictive performance of our method at predicting potential drug–ADR interactions that cause serious clinical outcomes. Figure 3a, b shows the AUCs and AUPRs of GCAP based on 10$\times$10-fold CV in the benchmark dataset, respectively. We observe that the GCAP obtains highly accurate diagnostic performance in 10 × 10-fold CV; s.t.d. AUC = 0.956 + −0.0011, and s.t.d. AUPR = 0.946 + −00017. Furthermore, two independent post-marketing test sets from Galeano’s study are collected^30^: (i) the SIDER test set containing 9387 drug–ADR interactions, and (ii) the OFFSIDES test set including 36,032 drug–ADR interactions (see Supplementary Data 3 and 4). After removing overlapping drugs from the benchmark dataset, 1330 and 4771 drug–ADR interactions are obtained from the two independent datasets, respectively. Figure 3c, d shows that our method gets 0.945 of AUC and 0.908 of AUPR in the SIDER test set and 0.937 of AUC and 0.931 of AUPR in the OFFSIDES test set, respectively. Moreover, we compare the classification performance of GCAP with that of other state-of-the-art baseline methods, including a matrix fcotrization method developed for drug-side effect frequency prediction^30^ and a deep-learning-based model for ADR prediction^31^. All the prediction methods were evaluated on a benchmark data set through cross-validation. To help readers estimate the difficulty of our task, we also report the performance of several machine-learning baseline methods in Supplementary Table 1.

We also evaluate the predictive performance of our method in determining the classes of serious clinical outcomes for drug–ADR interactions. Figure 3a, b shows the AUCs and AUPRs for each of the seven classes of serious clinical outcomes in the benchmark dataset based on the 10×10-fold CV, respectively. The AUCs of GCAP for the classes of serious clinical outcomes are 0.917 (DE), 0.859 (LT), 0.896 (HO), 0.932 (DS), 0.953 (CA), 0.948 (RI), and 0.916 (OT) and the AUPRs of GCAP for the classes of serious clinical outcomes are 0.806 (DE), 0.733 (LT), 0.849 (HO), 0.769 (DS), 0.529 (CA), 0.431 (RI), 0.688 (OT) (s.t.d. average AUC = 0.917$\pm$0.0009, and s.t.d. average AUPR = 0.687$\pm$00058). Figure 3c, d shows our method gets 0.891 on average AUC and 0.674 on average AUPR in the SIDER test set and 0.901 on average AUC and 0.663 on average AUPR in the OFFSIDES test set, respectively. The AUPR value of each class of serious clinical outcomes indicates that our method has achieved lower predictive performance in the CA and RI classes compared to the others which can be attributed to the small number of known drug–ADR interactions in these two classes. The detail of performance of GCAP in predicting the serious clinical outcomes’ class of adverse reactions to drugs are also listed in Supplementary Table 2. In addition, the method’s performance is robust with respect to the setting of the hyperparameters (Supplementary Tables 3–8).

Furthermore, several techniques have been introduced to overcome the challenges of missing or noisy data and improve the predictive performance. Comprehensive ablation studies have been conducted to demonstrate the importance of individual components of GCAP (see Supplementary Table 9). The first one is to apply optimized CNN and GNN modules to extract the potential representations of drugs from SMILES sequences. The predictive performance is improved by 1.5% and 3.6% in terms of average AUC and average AUPR on the classes of serious clinical outcomes for drug–ADR interactions, respectively. The second is to apply semantic descriptors and construct DAG for each ADR. The prediction performance of the GCAP on both tasks is improved after adding the *MLP*_*semantic*_ module. Besides, we find that the implementation of known drug–ADR interactions that cause serious clinical outcomes also contributes to improving predictive performance. The GCAP gain of 3.5% and 3.7% improvement in terms of AUC and AUPR on the binary classification task and 5.2% and 5.8% in terms of average AUC and AUPR on the second task, respectively. We further introduce the multi-head self-attention module to combine the features from multimodal data. The test result shows that the predictive performance improves 3.6% in terms of average AUPR on the second task of GCAP. These results indicate that the current model architecture and feature selection scheme are optimal for our prediction tasks, and GCAP serves as a powerful and robust framework for predicting the seriousness of clinical outcomes of adverse reactions to drugs.

### Performance evaluation on the new drugs and ADRs

**Fig. 4:**
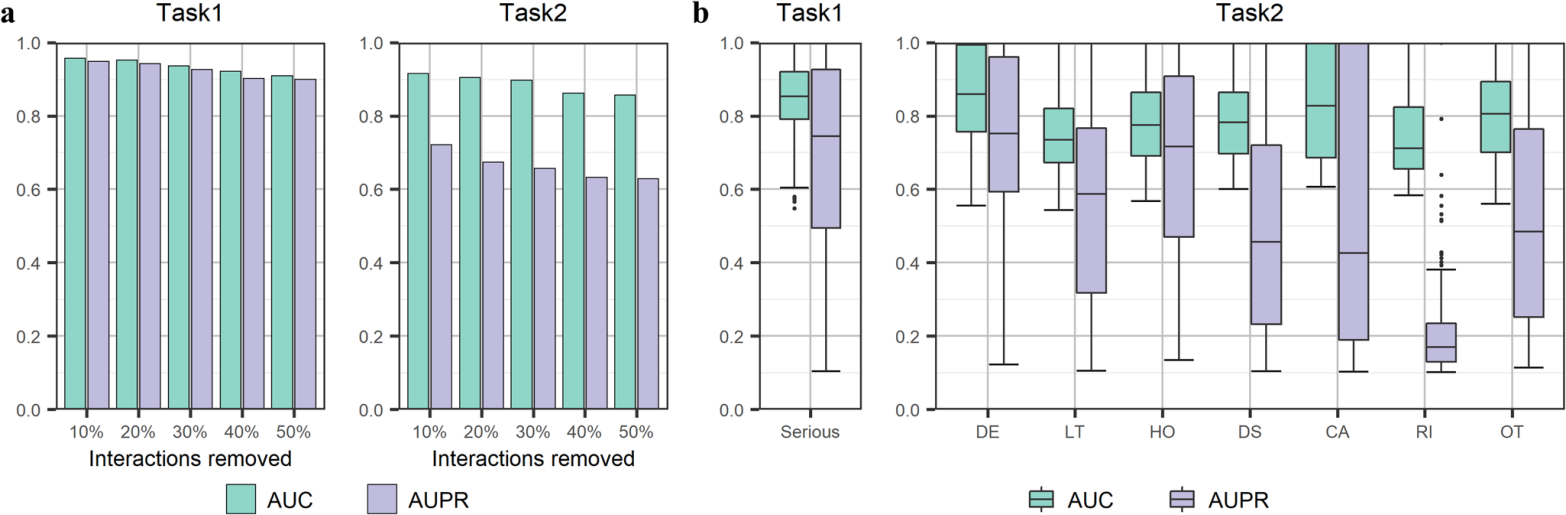
Evaluation of our method on the new drugs. **a** Mean AUC and AUPR at predicting the drug–ADR interactions that cause serious clinical outcomes and the corresponding classes of serious clinical outcomes for varying percentages of randomly chosen interactions. **b** The distribution of AUCs and AUPRs of drugs in de novo test. Since some drugs only have one or more of the seven seriousness classes in the benchmark dataset, it is not possible to obtain all the seriousness class labels. The boxes inside the violin plots indicate the 50-th percentile (middle line), 25-th and 75-th percentile (box), the smallest value within 1.5 times interquartile range below the 25-th percentile, and the largest value within 1.5 times interquartile range above the 75-th percentile (whiskers), and outliers (dots). We set the AUC and AUPR values of the corresponding seriousness classes of these samples to 0 and do not count them.

A key question for the real applicability of GCAP concerns its ability to predict the serious clinical outcomes of adverse reactions for new drugs and uncover serious clinical outcomes of new adverse reactions for approved drugs. With the deepening of clinical research and the continuous updating of market feedback information, the adverse reactions of approved drugs are gradually discovered in later clinical practice. Some serious clinical outcomes caused by adverse reactions to drugs are not noticed until years after they were on the market^32^. Here, we simulate the incremental discovery process by gradually and randomly removing a certain percentage of associated ADRs for each drug. More specifically, for each drug, if the number of the known drug-related interactions in the benchmark dataset is greater than 10, we remove a random 10% of these interactions to construct a testing set. We take all removed samples as the test set and the remaining samples in the benchmark dataset as the training set. We test the predictive performance of the model by gradually removing the number of ADRs associated with each drug in the training set. Figure 4a shows the AUCs and AUPRs of GCAP on the task of identifying whether the adverse reactions to drugs cause serious clinical outcomes in the context of different ratios of known drug–ADR interactions. The AUC only drops by 4.69% when 50% of the interactions for each drug are removed, indicating that GCAP remains reliable even when there is a reduction in known drug–ADR interactions. Similarly, Fig. 4b demonstrates that GCAP maintains its predictive performance for the seriousness classes prediction task when the known classes of serious clinical outcomes caused by drug–ADR interactions are rare. The results also show that removing 10% of the interactions from each drug yields similar results to those obtained by removing 10% of interactions from the benchmark dataset randomly, as presented in Fig. 3a. In summary, GCAP demonstrates reliability and robustness in predicting the seriousness of clinical outcomes of adverse reactions even when the available data is limited or partially missing. This makes it a potentially valuable tool for identifying serious clinical outcomes of new drugs or uncovering serious clinical outcomes of new adverse reactions for approved drugs.

Furthermore, we simulate the discovery process of adverse reactions to new drugs by removing all known ADRs of each test drug (de novo test). The de novo test is designed to explore the performance of the model under the scenario of cold start. In the scenario of the cold start for drugs, we enable the drugs in the test set to be all unknown to the training set. More specifically, in the de novo test, each drug in the benchmark dataset is left out in turn as the test sample and all known interactions between the drug and all existing ADRs are deleted. By predicting interactions that cause serious clinical outcomes and the corresponding class scores of serious clinical outcomes between the test drug and all ADRs in the benchmark dataset, the AUC and AUPR values are calculated for each drug, as shown in Fig. 4. For the first task (binary classification to identify the drug–ADR interactions that cause serious clinical outcomes), the AUC of all drug predictions ranges from 56.5% to 100% and the AUPR is from 10.4% to 100%. For the second task (multi-label classification to identify classes of serious clinical outcomes caused by drug–ADR interactions), the AUC of all drug predictions ranges from 54.3% to 100% while the AUPR ranges from 10.4% to 100%. These results suggest that GCAP does not fully rely on known drug–ADR interactions and can effectively extract information from SMILES sequences and semantic feature vectors of ADRs. By generating effective representations for input drugs and ADRs, GCAP demonstrates its potential in predicting serious clinical outcomes of adverse reactions to new drugs even in a cold start scenario.

### Identifying the serious clinical outcomes of Confusional State (CS) for Oxycodone and its analogs

Confusional State (CS) is a mental state characterized by bewilderment, emotional disturbance, lack of clear thinking, and perceptual disorientation^33^. We next investigate whether GCAP is able to correctly identify the classes of serious clinical outcomes of the interactions between Oxycodone (a known semisynthetic opioid analgesic) with its analogs and CS. In our benchmark dataset, there are five Oxycodone-analogous drugs associated with CS and four of them have drug-CS interactions that can result in serious treatment outcomes. To avoid “easy prediction”, we remove all known information related to drugs that shared similar SMILES sequences (defined as >90% SMILES sequence similarity) with Oxycodone (e.g., Hydrocodone and Naltrexone) from the training set. After removing these records and the information about the five Oxycodone-analogous drugs, we re-train the GCAP model. The five Oxycodone-analogous drugs are then combined with the 893 ADRs to construct an independent test set containing 4465 candidate interactions.

**Fig. 5:**
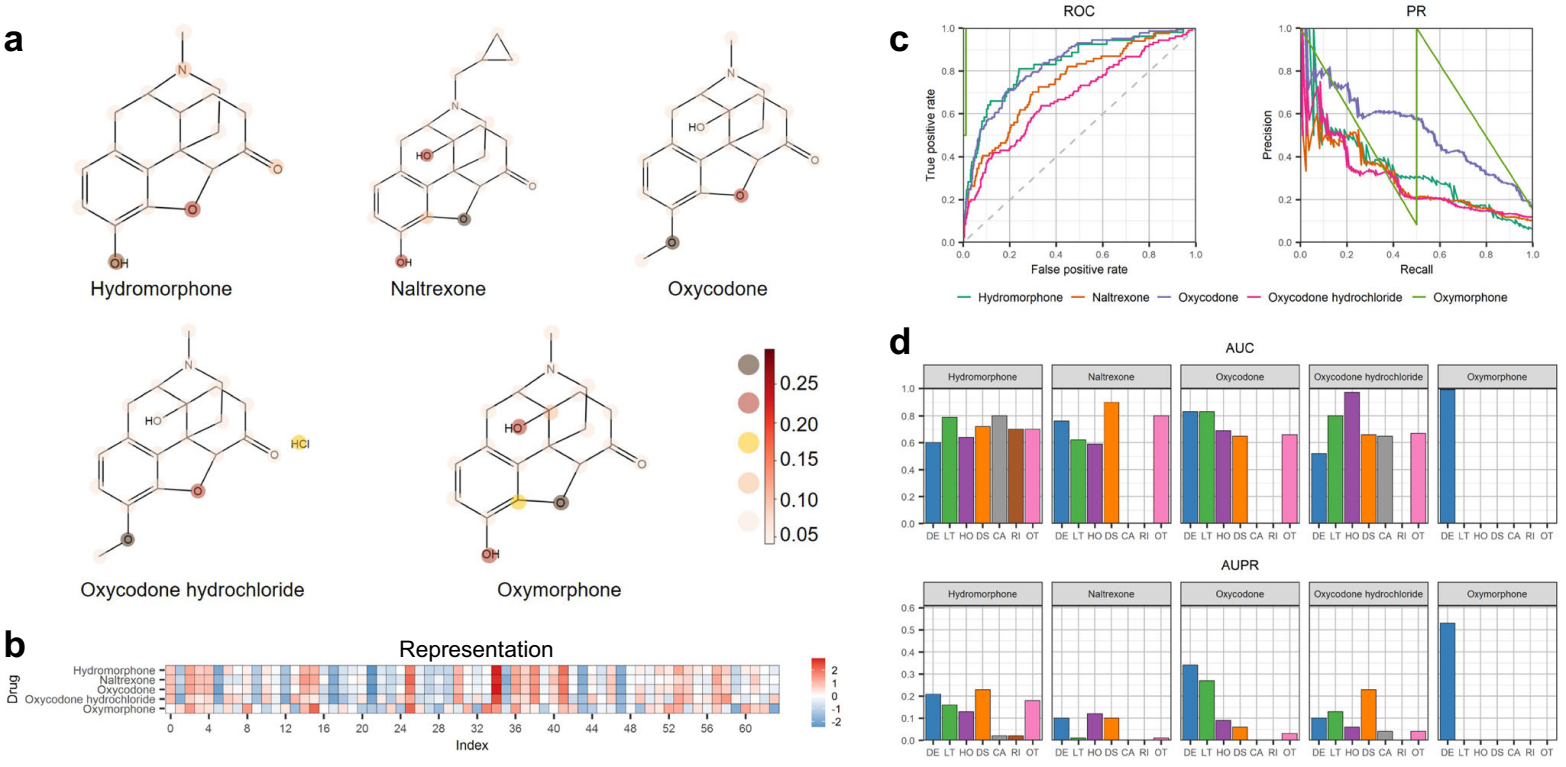
The performance of GCAP on Oxycodone-analog drugs. **a** The differences of attention scores of the MGA module for each atom in the molecular graph between five Oxycodone-analogous drugs interacting with CS. The darker the color is, the higher the attention value at the corresponding position is. **b** The differences of representations calculated by the MGA module between five Oxycodone-analogous drugs interacting with CS. **c**, **d** These show the predictive performance of GCAP for predicting the seriousness of adverse reactions to five Oxycodone-analogous drugs. Due to the fact that the clinical outcomes of some Oxycodone-analogous drugs related ADRs do not have all seven seriousness classes in the benchmark dataset, the AUC and AUPR of some Oxycodone-analogous drugs in the figure are zeros on the determination task of the class of serious clinical outcomes caused by drug–ADR interactions.

For the purpose of accurately representing drug molecular graphs, the GNN module assigns varying importance to each edge through the utilization of attention scores. In our study, we visualized the attention scores of the supernodes in the MGA module of GCAP for each atom in the five oxycodone drugs, along with the vector representations of the molecular graphs obtained through the MGA module. Figure 5a illustrates the attention scores of the super nodes in the MGA module of GCAP for the five Oxycodone-analogous drugs. Notably, for drugs with structural similarities, the atoms and substructures of interest for MGA exhibit remarkable similarity. Furthermore, Fig. 5b shows the representations generated by the MGA module of GCAP for the five Oxycodone-analogous drugs. Despite the strong similarity in the SMILES structures of these five Oxycodone-analogous drugs, the representations of samples with different labels (Hydromorphone, Naltrexone, Oxycodone, and Oxycodone hydrochloride labeled as 1, and Oxymorphone labeled as 0 in the benchmark dataset) exhibit differences. While the overall distributions of representations for analogs with the same label appear similar, certain positions (such as the 7th and 52nd positions) demonstrate distinct patterns. This discrepancy may arise from the models assigning different attention weights to atoms in the molecular graph. The MGA module’s output representation is obtained by aggregating the features of each atom in the molecular graph based on the corresponding attention scores.

The test results show that GCAP is able to correctly identify the relationships between Oxycodone-analog drugs and CS with an average AUC score of 0.816 and an average AUPR score of 0.398 (see Fig. 5c). (More details can be found in Supplementary Tables 10, 11, and 12). Such results further demonstrated the strong predictive power of GCAP. We also examine the predictive performance of GCAP for the classes of serious clinical outcome of adverse reactions to Oxycodone-analog drugs as shown in Fig. 5d. GCAP correctly identified most of the true seriousness classes of clinical outcome of the drug–ADR interactions with an average AUC of 0.732. These highly accurate predictions can provide valuable risk estimation for pharmacologists when considering the treatment of a patient with Oxycodone or its analogs (e.g., assessing the safety of Oxycodone).

### Extending the application of clinical outcomes of ADRs in three drug-related tasks

Zhang et al. ^34^ have shown that ADRs are predictive of drug–drug interactions. More recently, Zhou et al. ^35^ have shown that ADRs are predictive of drug response prediction. Therefore, one interesting question is whether our collected known drug–ADR interactions that cause serious clinical outcomes can be used as a feature to improve the predictive performance of existing models. To expore this, the potential application of the seriousness of clinical outcomes of ADRs is investigated in three drug-related tasks: drug–drug interaction prediction, drug response prediction, and drug side-effect frequency prediction. We observe the change in the predictive performance of some state-of-the-art methods for these three tasks after introducing the seriousness of clinical outcomes of adverse reactions to drugs to judge if it can be used as an effective representation of a drug. For a given model, the following steps are taken: (1) Collecting drugs that appear in both the model’s benchmark dataset and FAERS, and then reconstruct a new dataset based on these drugs; (2) Reproducing the prediction results from the model based on the new dataset using the code provided in the study, and (3) Building the model to obtain new prediction results by reasonably introducing the seriousness vectors of adverse reactions to drugs on a certain component of the model. The seriousness vectors for drugs are constructed based on the known drug–ADR interactions that cause serious clinical outcomes. The dimension of the vector is *m* (=893). If there is an interaction between the drug and the ADR in the benchmark dataset that causes serious clinical outcomes, the corresponding position is 1, otherwise, 0 (see Methods). By evaluating the performance of the two prediction results, the impact of the seriousness of clinical outcomes of drug–ADR interactions on the prediction performance of the model can be assessed. This will help determine whether the seriousness of clinical outcomes of ADRs can be used as an effective representation of a drug and contribute to enhancing the predictive performance of existing models in various drug-related tasks.

**Fig. 6:**
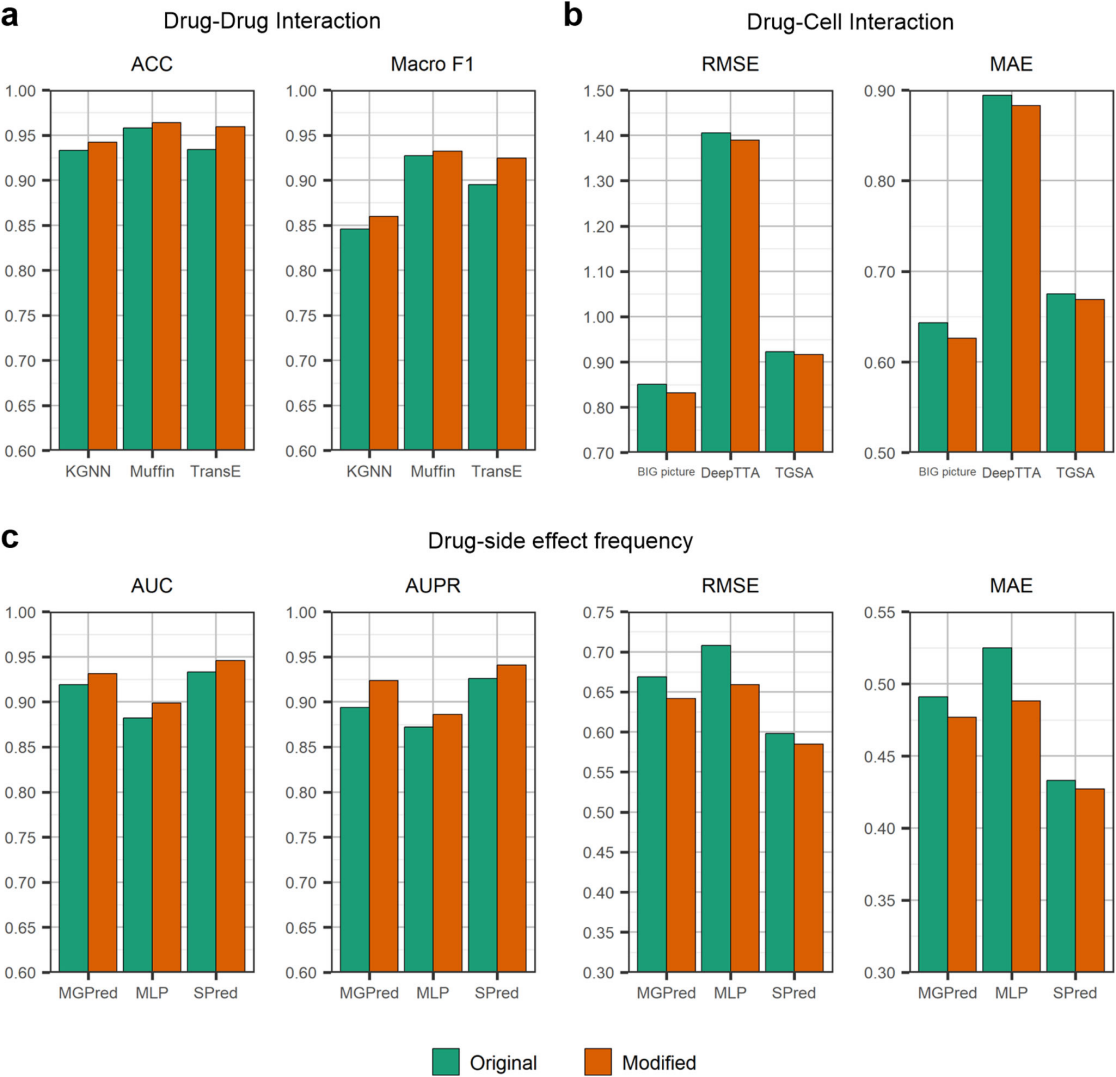
Seriousness of clinical outcomes of ADRs enables the state-of-the-art models to improve predictive performance under three drug-related tasks. Before and after the introduction of seriousness of clinical outcomes of adverse reactions to drugs, the prediction performance of the nine models changes. **a**, **b**, and **c** show the evaluation results of incorporating the seriousness vectors of ADRs into the models for drug–drug interaction prediction, drug-cell interaction prediction, and drug-side effect frequency prediction, respectively. All the evaluation metrics of the state-of-the-art models are obtained from their original papers.

We test three methods for the drug–drug interaction prediction task, including MUFFIN^36^, KGNN^37^, and TransE^38^ (see Fig. 6a). MUFFIN is a deep learning-based feature fusion framework for drug–drug interaction prediction. It can effectively integrate the features extracted from the drug’s molecular structure and knowledge graph. KGNN is an end-to-end framework that explores drugs’ topological structures in a knowledge graph for potential drug–drug interaction prediction. TransE is a knowledge graph representation method for learning low-dimensional embeddings of entities, which is often used as a baseline method for drug–drug interaction prediction. We test three methods for the drug response prediction task, including TGSA^39^, BIG picture^40^, and DeepTTA^41^ (see Fig. 6b). TGSA is a non-end-to-end deep learning method for drug response prediction, using twin graph neural networks and a similarity augmentation module. BIG picture is a method of using bipartite graphs for drug response prediction. DeepTTA is an end-to-end drug response prediction method that gets the representations of drugs through a transformer encoder and the representation of cell lines through four fully connected layers. For the drug side-effect frequency prediction task, we also test three methods, including MGPred^42^, SDPred^43^, and MLP (see Fig. 6c). MGPred is a deep learning framework to predict the side-effect frequencies of drugs by integrating chemical structure similarity, known drug side-effect frequency scores, side-effect semantic similarity, and pre-trained word representations. SDPred is a multi-task learning framework for drug–ADR interactions and frequencies by integrating the multi-correlation between the embedding of drugs and side-effects. MLP is a multi-layer perceptron with three hidden layers. The results in Fig. 6 show that all methods outperform the original method when the seriousness of clinical outcomes of adverse reactions to drugs is integrated as a feature. This strongly indicates that the seriousness of clinical outcomes of ADRs can be used as an effective representation to help solve drug-related problems. Further details of these methods are provided in Supplementary Note 2.

## Discussion

The correct prediction of the serious clinical outcomes caused by adverse reactions to drugs is critical to protecting patients and avoiding drug withdrawal from the market. In this work, we propose GCAP, a deep-learning framework for predicting the seriousness of adverse reactions to drugs, including the potential drug-–ADR interactions that cause serious clinical outcomes prediction and the corresponding classes of serious clinical outcomes determination. We first design optimal modules to extract the representation from SMILES sequences of drugs and MLP to extract the representation from DAGs of ADRs. Then, we stack all representations and feed them into a Fusion module with a multi-head attention mechanism to obtain the interactive representation between drugs and ADRs to enhance the predictive performance. Cross-validation and independent set test demonstrate that GCAP achieves satisfactory performance on both tasks. De novo test shows that GCAP maintains good predictive performance even when relying solely on SMILES sequences of drugs and semantic feature vectors of ADRs, indicating that the model is not entirely dependent on known drug–ADR interactions. This ability to make predictions for a broader range of drugs and ADRs expands the potential applications of GCAP. We also present five representative cases to visualize the attention weight results of the drugs and examine the predicted serious clinical outcomes of ADRs to Oxycodone and its analogs. Overall, the results demonstrate that GCAP can provide accurate seriousness predictions of clinical outcomes for adverse reactions to drugs, making it a valuable tool in protecting patients and preventing drug withdrawals from the market.

Earlier methods could predict drug–ADR interactions or their probabilities, but those probabilities did not indicate the classes of serious clinical outcomes for ADRs. By collecting treatment outcomes of serious adverse events from FAERS and defining the classes of serious clinical outcomes based on statistical methods, this work has developed a more comprehensive approach. Moreover, Fig. 6 demonstrates the prediction results of some state-of-the-art models for drug-related tasks, such as drug–drug interaction prediction, drug response prediction, and drug side-effect frequency prediction, after incorporating known drug–ADR interactions that cause serious clinical outcomes. The evaluation indicators reveal that the introduction of seriousness features of adverse reactions based on clinical outcomes helps improve the prediction performance of these models. This indicates that exploring the clinical outcomes of adverse reactions to drugs is valuable for researchers in formulating biological hypotheses related to drugs, side effects, and molecular mechanisms.

Despite its merits, the current version of GCAP still has some limitations. For example, GCAP only considers a single type of data, such as semantic vectors for ADRs and SMILES sequences for drugs, is learned as input. In practice, drugs and targets have different representations from different levels. The incorporation and fusion of heterogeneous information, such as drug targets^44^, therapeutic indications^45^, and perturbation transcriptomics data, could potentially enhance the performance of our tasks. We extract drug-target interactions from the latest version of the Drugbank database, identifying 582 drugs in the benchmark dataset that have at least one target. Using these drugs, we construct a new benchmark dataset and generate target feature vectors for each drug. The target vectors for drugs are derived from the known drug-target interactions recorded in Drugbank database. In total, Drugbank database contains 1374 targets associated with the 582 drugs present in our new benchmark dataset. For constructing the target feature vectors, if a drug and target have an interaction recorded in the Drugbank dataset, the corresponding position in the target vector is assigned a value of 1; otherwise, it is set to 0. The results of the tenfold cross-validation experiment revealed that incorporating the target vector as a feature of the drug led to a slight improvement in the predictive performance of the model (see Supplementary Table 13). Moreover, public databases have inherent limitations and biases. We observed that the reported classes of serious clinical outcomes for drug–ADR interactions in the benchmark dataset are skewed towards DE (see Fig. 1b). In the future, we plan to focus on gathering additional metadata from clinical trials and integrating multi-view representations in deep learning-based models to improve the model’s predictive accuracy.

## Methods

### The benchmark dataset

We construct a benchmark dataset from two sources, i.e., known drug–ADR interactions from ADReCS database version v3.1 (http://bioinf.xmu.edu.cn/ADReCS) and adverse event reports from United States (U.S.). Food and drug administration Adverse Event Reporting System (FAERS, https://open.fda.gov/data/faers/). The ADReCS database stores a large number of drug–ADR interactions extracted from DailyMed (http://dailymed.nlm.nih.gov/dailymed/about.cfm), which is a website managed by the U.S. National Library of Medicine to provide comprehensive information about marketed drugs. Moreover, ADReCS references MedDRA^46^ and UMLS^47^ for ADR term standardization, assigning each ADR a unique numerical ID that indicates its position in the ADR hierarchy tree and its linkage to other ADR terms. In this case, we extracted the known interactions between all drugs and the Preferred Terms (PTs) of ADRs. FAERS collects voluntary adverse drug reaction reports from healthcare professionals (e.g., doctors, pharmacists, nurses, etc.), consumers (e.g., patients, family members, attorneys, etc.), and clinical reviewers. The reports include the patient demographic and administrative information, drug/biologic information, adverse reaction information, and the patient treatment outcomes for the adverse events.

We collect the primary suspect drug, ADRs, and its corresponding treatment outcomes in each adverse event report. It is worth noting that treatment outcomes in FAERS are divided into seven classes: DEath (DE), Life-Threatening (LT), HOspitalization-Initial or prolonged (HO), DiSability (DS), Congenital Anomaly (CA), Required intervention to prevent permanent impairment/damage (RI), and OTher (OT). Based on these seven different treatment outcomes, we define seven distinct seriousness classes (OT = 1, RI = 2, CA = 3, DS = 4, HO = 5, LT = 6, DE = 7). We gather all adverse event reports in FAERS from the third quarter of 2014 to the first quarter of 2022 and determine the seriousness of clinical outcomes of known drug–ADR interactions using the Proportional Reporting Rate (PRR) approach:

1 $${{PRR}}_{{d}_{i}-{s}_{j},k}=\frac{{N}_{{d}_{i}-{s}_{j},k}/{N}_{{d}_{i}-{s}_{j},+}}{({N}_{+,k}-{N}_{{d}_{i}-{s}_{j},k})/({N}_{+,+}-{N}_{{d}_{i}-{s}_{j},+})},$$

where ${d}_{i}-{s}_{j}$ represents a drug–ADR interaction that appears in ADReCS and simultaneously the drug *d*_*i*_ is the primary suspect drug and appears in a record of FAERS with ADR *s*_*j*_, *k* represents a treatment outcome that appears in FAERS. ${N}_{{d}_{i}-{s}_{j},+}$ represents the number of the simultaneous occurrence of primary suspect drug *d*_*i*_ and ADR *s*_*j*_ in a record of FAERS, ${N}_{{d}_{i}-{s}_{j},k}$ represents the number of the simultaneous occurrence of suspect drug *d*_*i*_ and ADR *s*_*j*_ with the treatment outcome *k* in a record of FAERS, ${N}_{+,k}$ represents the number of records that the treatment outcome is *k* and ${N}_{+,+}$ represents the number of all records. The formula for calculating the 95% confidence interval for PRR is:

2 $${CI}\left({{PRR}}_{{d}_{i}-{s}_{j},k}\right)={e}^{{{{{{\rm{ln}}}}}}\left({{PRR}}_{{d}_{i}-{s}_{j},k}\right)\pm 1.96\times {Standard}{Error}({SE})},$$

where SE = $\sqrt{\frac{1}{{N}_{i-j,k}}-\frac{1}{{N}_{i-j,+}}+\frac{1}{{N}_{+,k}-{N}_{i-j,k}}+\frac{1}{{N}_{+,+}-{N}_{i-j,+}}}$. When the value of ${CI}\big({{PRR}}_{{d}_{i}-{s}_{j},k}\big)$ is greater than 1 (significant), we consider the clinical outcomes of drug–ADR interaction ${d}_{i}-{s}_{j}$ to be serious, and its seriousness class is k. Moreover, to maintain the high quality of the constructed dataset, we count the number of drugs associated with ADRs in ADReCS and select ADRs with >50 associated drugs. In total, the benchmark dataset contains a total of 141,752 drug–ADR interactions, covering 1073 drugs and 893 ADRs. Among these, 58,429 drug–ADR interactions can cause serious clinical outcomes and have corresponding seriousness class labels. The seriousness class label for each drug–ADR interaction that causes serious clinical outcomes is represented by a 7-dimensional vector. If a drug–ADR interaction has some seriousness class labels, the corresponding position on this vector is 1, and otherwise 0. Each drug in our dataset has a known SMILES sequence. A detailed explanation of the data processing is presented in Supplementary Note 1.

For convenience, let *n* (=1073) and *m* (=893) be the number of different drugs and ADRs in the benchmark dataset, respectively. Then we can construct an *n* $\times$ *m* matrix *R*_*Seriousness*_ to represent the interactions that cause serious clinical outcomes between drugs and ADRs in the benchmark dataset. The values of *R*_*Seriousness*_ are encoded with integers between 0 and 1. If a drug–ADR interaction matches the record in the benchmark dataset and causes serious clinical outcomes, then the value of the element at the corresponding position of matrix *R*_*Seriousness*_ is set to 1. Similarly, we can construct an *n* $\times$ *m* matrix *R*_*interaction*_ to represent all the drugs–ADRs interactions in the benchmark dataset. If the drug–ADR interaction appears in the benchmark dataset, the value of the element at the corresponding position of the matrix *R*_*interaction*_ is set to 1. The remaining entries in *R*_*Seriousness*_ and *R*_*Interaction*_ are filled with zeros.

### Drug molecule graph

Each drug possesses a unique chemical structure that is naturally represented by its molecular graph^48^. Here, we retrieve drug SMILES sequences from ADReCS and PubChem databases. Then, we use the DGL package^49^, a high-performance and scalable Python package for deep learning on graphs, to contract molecular graphs for drugs based on the SMILES sequence. A detailed explanation of how this is computed can be found in the DGL book in the documentation online (https://www.dgl.ai/). Nodes in a molecular graph are composed of chemical atoms of a drug, and molecular diagrams postulate that key interactions between nuclei and electrons in molecules can be implicitly captured by a diagram that provides a source of insight into the geometry, function, and properties of molecules^50,51^. We follow an approach similar to the one used by Cami et al. study^52^ to encode the initial features of bonds and atoms, and to unify the length of the vectors through linear transformations and nonlinear activations. Then, we use GNN and the attention mechanism to learn the representation of each atom by integrating its neighboring atom features.

### SMILES sequence encoding matrix

We use one-hot vectors to encode SMILES sequences and concatenate these vectors into a matrix to represent inputs of the CNN module. We collect SMILES sequences from PubChem and compiled 63 one-hot vectors for all SMILES sequences. Here, each atom, chemical bond, and parenthesis in the sequence is represented by a different one-hot vector. Considering that the length of SMILES sequences for different drugs may vary. We set the maximum length of SMILES to be 100, and SMILES sequences with length less than 100 are padded with zeros. For example, a SMILES sequence containing 20 elements is encoded in a 100 $\times$ 63 matrix. The first 20 rows of the matrix are composed of corresponding one-hot vectors, while rows 21 to 100 are all padded with zeros. We choose the maximum length of drugs to cover at least 80% of the intact drugs in our benchmark dataset and adopt the truncating strategy from a previous study^53^. SMILES sequences longer than the maximum length are truncated and those shorter than the maximum length are zero-padded.

### Semantic feature vectors of ADRs

The semantic features of ADRs have been widely applied to the drug side-effect prediction task and its effective performance has been fully demonstrated in plenty of previous studies^54,55^. Here, we calculate the semantic feature vectors of ADRs based on ADR descriptors in the ADReCS database. Each ADR descriptor in ADReCS is represented by a unique numerical ID indicating its position in the semantic hierarchy tree and its relationship to other ADR descriptors. A numeric ID is a combination of four fixed-length numeric strings, which represent the four levels of the ADReCS hierarchy from left to right. The more digital strings contained in the digital ID, the more specific the information indicated by the ADR descriptor. By correlating the associated descriptors of ADRs, we can construct a direct acyclic graph (DAG) for each ADR. Figure 2 displays the DAG of the ADR “Meningitis Bacterial” with the ADR descriptor “17.06.10.002; 11.02.01.013”. The number of all ADR descriptors related to it is 7. Different semantic descriptors may be associated with different sets of semantic descriptors. To capture this information, we count all possible semantic items of ADRs at each semantic level in the benchmark dataset and encode them as integers. The dimension of the semantic feature vector for ADRs matches the number of semantic items. If the DAG of an ADR contains a semantic term (id), the corresponding position of the vector is 1, otherwise, it is set to 0. Finally, we encode the semantic categories for each ADR in our study into a 792-dimensional multi-hot vector.

### Learning latent representations for drug–ADR interactions

It is well known that high quality feature encoding algorithms can capture key features of drugs and ADRs, helping improve prediction accuracy. In this work, we utilize a multi-level attention GNN module, a multi-scale residual CNN module, and a multi-head self-attention fusion module to extract the latent representations from input drugs and ADRs.

### Multi-level Graph Attention module

The intrinsic chemical structures of drugs and the GNN network have been employed to predict drug–target interactions^56^, drug–cell line interactions^57^, and anatomical therapeutic chemical codes of drugs^58^. Here, we use a Multi-level Graph Attention (MGA) module to learn the representation of the molecular graph. The MGA module introduces the self-attention mechanism^59^ in the propagation process of GNN layers, the core idea of which is to obtain the context vector of the target node by paying attention to the neighbors and local environment of the target node. MGA in GACP contains two attention mechanisms: an atomic-level attention machine and a molecular-level attention mechanism. In the atomic-level attention mechanism, each atom in a molecular graph progressively aggregates information from its neighborhood, focusing on the most relevant information in its neighborhood. In each node-embedded attention layer, a new state vector is generated for each atom. After several stacked attention layers, the state vector contains more neighborhood information. To integrate the vector of each atom in the molecular graph into the vector of the whole molecule, we employ the molecular-level attention mechanism, constructing a super node to represent the information of the entire molecular graph. The super node is connected to all nodes in the molecular graph. Operating similarly to the attention mechanism at the atomic level, the final representation of the entire drug molecular graph can be obtained. MGA not only characterizes the local atomic environment by broadcasting node information from nearby nodes to more distant nodes but also allows non-local effects at the intramolecular level by applying a graph attention mechanism.

To give readers a better understanding of the MGA process, we take a graph molecular *G* = (*V*, *E*) as an example and discuss the specific implementation details of each step.

In step 1, we update the node information by aggregating the information of the edges corresponding to the nodes in the graph. Firstly, we determine the contribution of each edge information to the nodes. For example, let ${v}_{i}\in V$ and ${e}_{j}\in E$, the contribution of *e*_*j*_ to *v*_*i*_ can be calculated as follows:

3 $${Z}_{i,j}={Relu}\left({W}_{1}\left[{h}_{{v}_{i}}^{{init}}\right|\left|{h}_{{e}_{j}}^{{init}}\right]\right),$$

where ${h}_{{v}_{i}}^{{init}}$ and ${h}_{{e}_{j}}^{{init}}$ are the initiation representations of *v*_*i*_ and *e*_*j*_, respectively, *W*_*1*_ is a transformer matrix, and Rectified Linear Unit (ReLU) function^60^ is the activation function. Then, we use the Softmax function^61^ to normalize the contribution of all edges associated to *v*_*i*_ to obtain the attention coefficient.

4 $${\alpha }_{i,j}={softmax}\left({Z}_{i,j}\right)=\frac{{{\exp }}({Z}_{i,j})}{{\sum }_{k\in {P}_{i}}{{\exp }}({Z}_{i,k})},$$

where *P*_*i*_ represents all edges associated with *v*_*i*_. Finally, we obtain the output representation of node *v*_*i*_ by weighting the features of the edges.

5 $${h}_{{v}_{i}}^{0}={Relu}({\sum }_{{e}_{j}\in {P}_{i}}{\alpha }_{i,j}{h}_{{e}_{j}}^{{init}})$$

${h}_{{v}_{i}}^{0}$ is fed into a Gated Recurrent Unit cell^62^ together with the target atom’s current state vector ${h}_{{v}_{i}}^{{init}}$, producing the updated state vector ${h}_{{v}_{i}}^{1}$ of atom *v*_*i*_.

In step 2, we update the node information by aggregating the information of all neighbor nodes (self-attention mechanism). The process of updating node information is similar to the method in step 1, except that this experiment uses the neighbor node information instead of the edge information to update node representation. Finally, we can get the updated state vector ${h}_{{v}_{i}}^{2}$ to represent the atom *v*_*i*_.

In step 3, we combine all atomic representations in the molecular graph into the representation of the entire molecule. We construct a super node *v*_*super*_ for the *G*, which is connected to all the nodes in the molecular graph, and its initial representation is the accumulation of all the nodes in the molecular graph. Then, we generate the representation of the super node using the same computational steps as in step 2. This process is carried out on a multilayer attention layer of molecular embedding and generates a representation vector for the whole molecular graph. In the final stage of the MGA module, we apply a fully connection layer over the representation to capture the most important feature and get the drug latent representation.

### Multi-scale residual CNN module

Inspired by the great success of CNN networks in natural language processing applications^63^, we propose a Multi-scale Residual CNN module to extract the latent information from SMILES sequences. The architecture is able to integrate local dependencies to capture latent representations of SMILES sequences. When the convolution filter slides on the SMILES coding matrix, the local dependencies between different substructures of the drug are captured by the latent feature vectors. More specifically, the CNN module in GCAP comprises multiple separate CNN layers and each layer contains multiple different 1-dimensional filters. In a CNN layer of GCAP, the fusion process is performed via the convolution operation and ReLU function. The initial feature input of the CNN module in GCAP is the SMILES encoding matrix encoded by the Pytorch embedding layer^64^. Let *x*_*i*_ represent the *i*-th row in the matrix. The input matrix of a CNN layer is represented as:

6 $${x}_{1:l}={x}_{1}\oplus {x}_{2}\ldots {x}_{l}$$

where ⊕ is the concatenation operator and *l* is the number of columns of the matrix. Then, multiple convolution filters of different scales and the ReLU function are applied to windows of the input matrix to produce new features. For example, a convolution kernel *w* with size *h* in the convolution operation is applied to a window of *h* rows of the input matrix to produce a new feature as follows:

7 $${c}_{i}={ReLU}(w{{{{{\rm{\cdot }}}}}}{x}_{i:i+h-1}+b)$$

where *b* is a bias term. By checking all possible windows in the ${x}_{1:l}$, we can obtain a feature map:

8 $${{{{{\rm{c}}}}}}=[{c}_{1},\,{c}_{2},\,.\,.\,{c}_{i},\,.\,..\,,\,{c}_{l}]$$

Here, we set the padding to [(*h*−1)/2] and stride to 1 to ensure that the input and output dimensions are consistent. We set up four filters to obtain multiple features. Each filter has *l* channels and their scale sizes are 1, 3, 5, and 7, respectively. Then, we concatenate all feature maps and use them as input to the next CNN layer. In addition, we add a residual path between two adjacent CNN layers to alleviate the problem of network degradation^65^. In the final stage of the CNN module, we apply a max-pooling operation over the feature maps to perform feature selection and get the drug latent representation.

### Representation fusion

We design a fusion module, a multi-head self-attention module, that fuses multiple representations into a joint vector for downstream predictions. More specifically, taking drug–ADR interaction *d*_*i*_-*s*_*j*_ as an example, we use the fully connected layers to extract the semantic feature vector of ADR *s*_*j*_, the *j*-th column of *R*_*Interaction*_ and *R*_*Seriousness*_ and perform feature extraction and dimension transformation to obtain representations ${f}_{{{{{{\rm{semantic}}}}}}}^{{ADR}}$, ${f}_{{{{{{\rm{interaction}}}}}}}^{{ADR}}$, and ${f}_{{{{{{\rm{seriousness}}}}}}}^{{ADR}}$ for *s*_*j*_ respectively. We use the fully connected layers, multi-level graph attention module and multi-scale residual CNN module for SMILES sequences, the *i*-th row of the known interaction matrix *R*_*Interaction*_ and known seriousness matirx *R*_*Seriousness*_ to obtain representations ${f}_{{{{{{\rm{GNN}}}}}}}^{{drug}}$, ${f}_{{{{{{\rm{CNN}}}}}}}^{{drug}}$, ${f}_{{{{{{\rm{interaction}}}}}}}^{{drug}}$, and ${f}_{{{{{{\rm{seriousness}}}}}}}^{{drug}}$ for *d*_*i*_ respectively. Then, we stack the seven representations into a matrix *M*, and perform the multi-head self-attention mechanism on these representations to ensure that each vector is aware of the fellow cross subspace representations. This operation enables each representation to induce latent information from other representations of the drug–ADR interaction. The single-headed attention mechanism is defined as a scaled dot-product function:

9 $${{Head}}_{i}={{{{{\rm{Attention}}}}}}\left({Q}_{i},{K}_{i},{V}_{i}\right)=\frac{{Softmax}({Q}_{i}{K}_{i}^{T})}{\sqrt{{L}_{e}}}{V}_{i}$$

where *Q*, *K*, *V* are the query, key, and value matrices of *head*_*i*_, which are obtained by multiplying the corresponding linear projection matrices (*W*_*Q*_, *W*_*K*_, and *W*_*V*_) with the input data, respectively. *T* is a transpose operation and ${L}_{e}$ is the dimensional of *W*_*Q*_, *W*_*K*_, and *W*_*V*._ The multi-head attention mechanism computes multiple such single-head attentions in parallel, and then concatenates the results of each single-head attention to obtain the final result. The calculation process is defined as follows:

10 $${{{{{\rm{Multi}}}}}}-{{{{{\rm{head}}}}}}={{Head}}_{1}\oplus {{Head}}_{2}\oplus \ldots {{Head}}_{i}\oplus \ldots {{Head}}_{R}$$

where ⊕ is the concatenation operator and *R* is the number of heads in the multi-head attention mechanism. Finally, we perform a pooling operation on the output of the fusion module to generate the final representation of the *d*_*i*_–*s*_*j*_ interaction.

### Predicting the seriousness of clinical outcomes of adverse reactions to drugs

Our model has two subtasks, the first one focuses on identifying serious/non-serious clinical outcomes of adverse reactions to drugs, and the second one focuses on predicting the classes of serious clinical outcomes caused by drug–ADR interactions. Therefore, we set up two separate MLP modules to obtain GCAP prediction results. Each MLP module has a fully connected hidden layer and the same number of neurons. The first MLP module is predicting the seriousness scores of clinical outcomes between the drugs and ADRs and the second MLP module is used to predict the seriousness class scores between the drugs and ADRs.

### Multi-objective optimization

The goal of GCAP is to minimize the difference between the predicted scores and the true seriousness labels of known drug–ADR interactions. Therefore, GCAP has two separate loss functions including a binary cross-entropy loss function and a diversity cross-entropy loss function, for the corresponding two tasks, respectively. For the serious/non-serious clinical outcomes prediction task, in a training set with *N* drug–ADR interactions, the binary cross-entropy loss function is defined as:

11 $${{Loss}}_{{association}}=-\frac{1}{N}\mathop{\sum }\limits_{i=1}^{N}{y}_{i}^{{true}}\times {{\log }}\left({y}_{i}^{{pred}}\right)+(1-{y}_{i}^{{true}})\times {{\log }}\left(\left(1-{y}_{i}^{{pred}}\right)\right)$$

where *N* is the number of samples in the training set, ${y}_{i}^{{pred}}$ and ${y}_{i}^{{true}}$ represent the predictive score and true label of the *i*-th sample, respectively.

For the task of determining the classes of serious clinical outcomes caused by drug–ADR interaction, the final diversity cross-entropy loss function is defined as:

12 $${{Loss}}_{{Severity}}=	 -\frac{1}{{C\times N}_{i}}\mathop{\sum }\limits_{i=1}^{C}\mathop{\sum }\limits_{j=1}^{{N}_{i}}{y}_{i,j}^{{true}}\times {{\log }}\left({y}_{i,j}^{{pred}}\right)\\ 	 +(1-{y}_{i,j}^{{true}})\times {{\log }}\left(1-{y}_{i,j}^{{pred}}\right)$$

where *C* is the total number of classes, *N*_*i*_ is the number of samples belonging to the *i*-th class in the training set, *y*_*ij*_ is the label of the *j*-th sample belonging to the *i*-th class. The format of the labels is represented by a multi-hot vector and ${y}_{i,j}^{{pred}}$ represents the predicted probability of the *j*-th sample belonging to the *i*-th class. The two loss functions are combined together and optimized simultaneously in a multi-objective training process as follows:

13 $${{Loss}}_{{total}}={{Loss}}_{{association}}+\alpha {{Loss}}_{{Severity}}$$

where $\alpha$ stands for a weight parameter that balances the two loss functions. All parameters of GCAP are updated using the Adam optimizer^66^. The training process is efficient, with a single GCAP model able to be trained within two hours on a Linux server with 20 logical CPU cores and one Nvidia GeForce GTX 2080Ti GPU.

### Evaluation protocols and metrics

To minimize the impact of data variability on the results, we use tenfold cross-validation to evaluate the predictive performances of our method. This validation process ensures that the model is tested on different subsets of the data, making the evaluation more robust and less prone to overfitting. In the *k*-th fold, the *k*-th positive and negative subsets are set as the testing set for model testing, and the remaining nine positive and negative subsets are set as the training set for model training. A higher rank for the positive samples indicates a better predictive performance of the method.

The predictive performance of the method is evaluated using the AUC and AUPR metrics. These metrics provide a comprehensive evaluation of the model’s performance across different thresholds. The ROC curve is an efficient indicator for visualizing and measuring the cost of the true positive rate (TPR) against the false positive rate (FPR) at various thresholds. The ROC curve is a useful indicator for visualizing and measuring the trade-off between the TPR and the FPR at various thresholds. A high AUC value indicates that the classifier is more likely to rank a randomly chosen positive instance higher than a randomly chosen negative instance, reflecting better predictive performance. The PR (Precision-Recall) curve demonstrates the trade-off between precision and recall for different thresholds. A high AUPR value indicates both high recall and precision, which means the model is able to correctly identify a large proportion of positive instances while maintaining a low rate of false positives.

### Reporting summary

Further information on research design is available in the Nature Portfolio Reporting Summary linked to this article.

### Supplementary information

Supplementary Information

Description of Additional Supplementary Files

Supplementary Data 1

Supplementary Data 2

Supplementary Data 3

Supplementary Data 4

Supplementary Data 5

Supplementary Data 6

Reporting Summary

## Data Availability

Data availability are as follows: All raw known drug–ADR interactions are collected from ADReCS database version v3.1 (http://bioinf.xmu.edu.cn/ADReCS/). All raw adverse event reports are collected from the united states Food and drug administration Adverse Event Reporting System (FAERS, https://open.fda.gov/data/faers/). drug–ADR interactions in the benchmark dataset (Supplementary Data 1), classes of serious clinical outcomes caused by drug–ADR interaction in the benchmark dataset (Supplementary Data 2), the SMILES sequences of drugs in the benchmark dataset (Supplementary Data 3), the semantic descriptions of ADRs in the benchmark dataset (Supplementary Data 4), and two independent test datasets (Supplementary Data 5 and 6). Source data for figures can be found in https://github.com/zhc940702/GCAP. Any other relevant data are available from the authors upon reasonable request.

## Code availability

The code of our deep learning model is provided in https://github.com/zhc940702/GCAP and Zendo^67^.
