# Supplementary material for: Identifying the serious clinical outcomes of adverse reactions to drugs by a multi-task deep learning framework
Source: Commun Biol. 2023 Aug 24;6:870. doi: 10.1038/s42003-023-05243-w (PMC10449791; doi:10.1038/s42003-023-05243-w)
Supplement: Supplementary file 1 — Supplementary Information [file 42003_2023_5243_MOESM1_ESM.pdf]

Supplementary Information for

"Identifying the serious clinical outcomes of adverse reactions to drugs by a multi-task deep learning framework"

Supplementary Tables

Supplementary Table 1 The performance comparison of different methods on the benchmark dataset.

Model	Accuracy	Precision	Recall	F1 score	AUC	AUPR
Galeano's method[1]	0.827	0.899	0.736	0.811	0.926	0.903
Wang's method[2]	0.752	0.872	0.466	0.608	0.748	0.889
Our method	**0.883**	**0.904**	**0.802**	**0.851**	**0.956**	**0.946**

Supplementary Table 2 The performance of GCAP in predicting the serious clinical outcomes' class of adverse reactions to drugs.

Class	Accuracy	Precision	Recall	F1 score	AUC	AUPR
DE	0.872	0.812	0.646	0.720	0.917	0.806
LT	0.803	0.744	0.628	0.682	0.859	0.733
HO	0.821	0.813	0.761	0.786	0.896	0.849
DS	0.896	0.791	0.629	0.701	0.932	0.769
CA	0.992	0.95	0.377	0.54	0.953	0.529
RI	0.991	0.909	0.203	0.332	0.948	0.431
OT	0.899	0.781	0.508	0.615	0.916	0.688
Average	0.896	0.829	0.536	0.625	0.917	0.687

Supplementary Table 3 Performance comparison of GCAP with different dropout rate.

Dropout	Task 1		Task 2	
	AUC	AUPR	Average AUC	Average APR
0	0.937	0.922	0.871	0.601
0.1	0.947	0.936	0.901	0.654
0.2	0.950	0.938	0.909	0.678
**0.3**	0.957	0.947	0.918	0.689
0.4	0.952	0.945	0.912	0.661
0.5	0.946	0.944	0.896	0.659

Supplementary Table 4 Performance comparison of GCAP with different loss weight α.

α	Task 1		Task 2	
	AUC	AUPR	Average AUC	Average APR
0.01	0.953	0.944	0.805	0.454
0.1	0.954	0.945	0.882	0.601
**1**	0.957	0.947	0.918	0.689
10	0.951	0.940	0.906	0.669
100	0.939	0.925	0.901	0.656

Supplementary Table 5 Performance comparison of GCAP with different learning rate.

Learning rate	Task 1		Task 2	
	AUC	AUPR	Average AUC	Average APR
0.00001	0.955	0.945	0.901	0.648
**0.00005**	0.957	0.947	0.918	0.689
0.0001	0.950	0.940	0.910	0.685
0.0005	0.955	0.946	0.906	0.680
0.001	0.954	0.945	0.902	0.668
0.005	0.820	0.767	0.602	0.242

Supplementary Table 6 Performance comparison of GCAP with different CNN layer.

CNN layer	Task 1		Task 2	
	AUC	AUPR	Average AUC	Average APR
1	0.947	0.945	0.906	0.673
2	0.957	0.947	0.907	0.675
3	0.950	0.940	0.913	0.682
4	0.955	0.946	0.914	0.680
**5**	0.957	0.947	0.918	0.689
6	0.957	0.947	0.916	0.676
7	0.956	0.947	0.914	0.680

Supplementary Table 7 Performance comparison of GCAP with different GNN layer.

GNN layer	Task 1		Task 2	
	AUC	AUPR	Average AUC	Average APR
1	0.955	0.946	0.917	0.680
2	0.956	0.947	0.917	0.681
**3**	0.957	0.947	0.918	0.689
4	0.955	0.944	0.916	0.682
5	0.954	0.945	0.911	0.679
6	0.943	0.932	0.879	0.614

Supplementary Table 8 Performance comparison of GCAP with different multi-heads.

Attention head	Task 1		Task 2	
	AUC	AUPR	Average AUC	Average APR
2	0.955	0.948	0.912	0.670
4	0.957	0.947	0.915	0.675
8	0.956	0.947	0.916	0.678
16	0.957	0.946	0.916	0.679
**32**	0.957	0.947	0.918	0.689
64	0.957	0.948	0.914	0.689

Supplementary Table 9 Performance comparison of CGAP with six alternative versions through 10-fold cross-validation.

Remove module	Task 1		Task 2	
	AUC	AUPR	Average AUC	Average APR
MRCNN	0.952	0.942	0.909	0.688
MGA	0.956	0.946	0.912	0.673
MRCNN and MGA	0.950	0.943	0.904	0.665
Fusion	0.950	0.940	0.910	0.675
$MLP_{Semantic}$	0.955	0.946	0.906	0.680
$MLP_{Association}$	0.953	0.943	0.908	0.684
$MLP_{Severity}$	0.954	0.944	0.905	0.667
$MLP_{Association}$ and $MLP_{Severity}$	0.923	0.914	0.872	0.651

Supplementary Table 10 The severe association prediction scores and labels of confusional state (CS) when predicting the drugs of Oxycodone and its analogs.

Drug name	SMILES sequence	Label	Prediction score
Hydromorphone	CN1CCC23C4C1CC5=C2C(=C(C=C5)O)OC3C(=O)CC4	1	0.845
Naltrexone	C1CC1CN2CCC34C5C(=O)CCC3(C2CC6=C4C(=C(C=C6)O)O5)O	1	0.879
Oxycodone	CN1CCC23C4C(=O)CCC2(C1CC5=C3C(=C(C=C5)OC)O4)O	1	0.883
Oxycodone hydrochloride	CN1CCC23C4C(=O)CCC2(C1CC5=C3C(=C(C=C5)OC)O4)O.Cl	1	0.967
Oxymorphone	CN1CCC23C4C(=O)CCC2(C1CC5=C3C(=C(C=C5)O)O4)O	0	0.172

Supplementary Table 11 The severity class prediction labels of confusional state (CS) when predicting the drugs of Oxycodone and its analogs.

Drug name	Label						
	OT	CA	DS	HO	LT	LF	DE
Hydromorphone	0	0	0	0	0	0	1
Naltrexone	0	0	0	0	1	0	0
Oxycodone	0	0	0	0	1	0	0
Oxycodone hydrochloride	0	0	0	1	0	0	1
Oxymorphone	0	0	0	0	0	0	0

Supplementary Table 12 The severity class prediction scores of confusional state (CS) when predicting the drugs of Oxycodone and its analogs.

Drug name	Prediction score						
	DE	LF	LT	HO	DS	CA	OT
Hydromorphone	0.142	0.351	0.505	0.116	0.003	0.001	0.005
Naltrexone	0.028	0.028	0.905	0.065	0	0	0.014
Oxycodone	0.095	0.234	0.833	0.066	0	0.001	0.003
Oxycodone hydrochloride	0.031	0.685	0.259	0.595	0	0.003	0.001
Oxymorphone	0.079	0.131	0.598	0.052	0.02	0.02	0.003

Supplementary Table 13 Impact of drug target features on predictive performance of GCAP.

Feature type	Task 1		Task 2	
	AUC	AUPR	Average AUC	Average APR
Original features	0.944	0.942	0.906	0.669
Original features + Drug target features	0.947	0.945	0.911	0.675

Supplementary Notes

Supplementary Note 1: The detailed description of the data processing procedure.

We initially extract the known interactions between all drugs and the Preferred Terms (PTs) of ADRs from ADReCS database. ADReCS follows a similar hierarchical structure as MedDRA and WHO-ART, consisting of four levels: System Organ Class (SOC), High-Level Group Term (HLGT), High-Level Term (HLT), and Preferred Term (PT). Each ADR term in ADReCS is assigned to this hierarchical tree, with increasing specificity from SOC to PT, where the PT represents a unique and unambiguous single ADR concept. To avoid redundancies in side effect terms, we specifically focus on the PT side effect terms in our analysis. This selection ensures that we capture specific and distinct ADR concepts. After performing the necessary data filtering steps, we obtain 467,854 drug-ADR interactions involving 2,377 drugs and 12,051 ADRs from ADReCS database. Next, we collect all serious ADR reports from the FAERS database, covering the period from the third quarter of 2014 to the first quarter of 2022. By matching the 'Primaryid' in each file from FAERS and removing incomplete records, we obtain a dataset of 6,873,533 serious ADR records. We then identify drugs and ADRs that co-occur in both ADReCS and FAERS databases and employ statistical methods to assess whether the interactions between these drugs and ADRs result in serious clinical outcomes. This analysis reveals 255,968 drug-ADR interactions that appear in the serious ADR records from FAERS. To determine the seriousness of clinical outcomes for these drug-ADR interactions, we apply the Proportional Reporting Rate (PRR) approach. Based on the PRR values, we assign seriousness labels to the drug-ADR interactions, classifying them as positive samples. Interactions related to drugs and ADRs present in ADReCS but not in FAERS are considered as negative samples. Following these operations, we obtain a final dataset comprising 189,779 drug-ADR interactions involving 1,175 drugs and 4,265 ADRs. To maintain the high quality of the constructed dataset, we apply a filtering criterion that considers the number of drugs associated with each ADR in ADReCS. Specifically, we select ADRs that are associated with more than 50 drugs, resulting in a benchmark dataset of 141,752 drug-ADR interactions. This dataset covers 1,073 drugs and 893 ADRs, ensuring a comprehensive and high-quality foundation for our analysis. Overall, the described process allows us to establish a robust dataset for studying drug-ADR interactions and their potential impact on serious clinical outcomes.

Supplementary Note 2: Some state-of-the-art methods for three drug-related tasks.

TGSA[3]: A non-end-to-end drug response prediction method based on deep learning. TGSA is composed of two major steps: twin Graph neural networks for Drug Response Prediction (TGDRP) and a Similarity Augmentation (SA) module. TGDRP learns drug features and cell line features through two identical graph neural networks respectively. SA utilizes GNNs to smooth the representations of similar cell lines/drugs. In this work, we add a fully connected layer to extract features from the ADR seriousness vectors of drugs and then accumulate it with the output of GNN_{drug} module in TGDRP.

BIG picture[4]: A drug response prediction method based on the bipartite graph. The bipartite graph is formed in two steps, selecting the most sensitive and most resistant cell lines for each drug and using known drug-cell line interaction to connect the drugs and the selected cell lines.

To obtain drug features, BIG picture learns node features on bipartite graphs through a heterogeneous graph convolution network (H-GCN). To obtain embeddings of the cell, BIG picture uses an independent multi-layer perceptron (MLP) module to extract features from gene expression data of cell lines. In our work, we add a fully connected layer to extract features from the ADR seriousness vectors of drugs and then accumulate it with the output of H-GCN module in BIG picture.

DeepTTA[5]: An end-to-end drug response prediction method based on a transformer encoder module. In DeepTTA, drugs firstly are divided into some substructure sequence vectors by ESPF. Then, these vectors are fed into the transformer encoder to get representations of the drugs. DeepTTA learns cell line features through an MLP module. In our work, we add a fully connected layer to extract features from the ADR seriousness vectors of drugs and then accumulate it with the output of the transformer encoder module in DeepTTA.

MUFFIN[6]: A deep learning-based feature fusion framework for binary-class, multi-class and multi-label drug-drug interaction prediction. It can effectively integrate the features extracted from the drug's molecular structure and knowledge graph. Here, we only evaluate the multi-class drug-drug interaction predictive performance of the model. We add a fully connected layer to extract features from the ADR seriousness vectors of drugs and let it be spliced with the other four feature vectors in the original paper and enter the classification module.

KGNN[7]: An end-to-end framework that explores drugs' topological structures in knowledge graph for potential drug-drug interaction prediction. By extending the receptive field of each entity in knowledge graph, KGNN is able to capture high-order relations between drug pairs. In our work, we add a fully connected layer to extract features from ADR seriousness vectors of drugs and then concatenate it with the latent representations of drugs output by the second step in KGNN layer to construct new representations of drugs.

TransE[8]: A knowledge graph representation method for learning low-dimensional embeddings of entities, which is often used as a baseline method for drug-drug interaction prediction. TransE interprets relationships between entities as translation vectors between head and tail entities on the low-dimensional embedding vector space. In our work, we add a fully connected layer to extract features from ADR seriousness vectors of drugs and then concatenate it with the latent representations of drugs output by TransE to construct new representations of drugs.

MGPred[9]: A deep learning framework to predict the side effect frequencies of drugs by integrating chemical structure similarity, known drug-side effect frequency scores, side effect semantic similarity, and pre-trained word vector representations. The core of the model is to construct a drug-side effect bipartite graph and learn the feature representations of the node in the graph from the node's direct neighbors based on the attention mechanism. In our work, we use ADR seriousness vectors of drugs instead of drug fingerprint-based vectors.

SDPred[10]: An end-to-end method based on multiple similarities and shares the advantages of both the matrix decomposition methods and deep learning methods and do not entirely

dependent on the known relationships between drugs and side effects. In our work, we calculate a new type of similarity for drugs based on the ADR seriousness vectors and extend the drug similarity types in SDPred to 11.

Supplementary References

1 Galeano, D., Li, S., Gerstein, M. & Paccanaro, A. Predicting the frequencies of drug side effects. *Nature communications* **11**, 1-14 (2020).

2 Wang, C.-S. *et al.* Detecting potential adverse drug reactions using a deep neural network model. *Journal of medical Internet research* **21**, e11016 (2019).

3 Zhu, Y. *et al.* TGSA: protein–protein association-based twin graph neural networks for drug response prediction with similarity augmentation. *Bioinformatics* **38**, 461-468 (2022).

4 Hostallero, D. E., Li, Y. & Emad, A. Looking at the BiG picture: incorporating bipartite graphs in drug response prediction. *Bioinformatics* **38**, 3609-3620 (2022).

5 Jiang, L. *et al.* DeepTTA: a transformer-based model for predicting cancer drug response. *Briefings in Bioinformatics* **23**, bbac100 (2022).

6 Chen, Y. *et al.* MUFFIN: multi-scale feature fusion for drug–drug interaction prediction. *Bioinformatics* **37**, 2651-2658 (2021).

7 Lin, X., Quan, Z., Wang, Z.-J., Ma, T. & Zeng, X. KGNN: Knowledge Graph Neural Network for Drug-Drug Interaction Prediction. *IJCAI2020.* 2739-2745 (2020).

8 Bordes, A., Usunier, N., Garcia-Duran, A., Weston, J. & Yakhnenko, O. Translating embeddings for modeling multi-relational data. *Advances in neural information processing systems* **26** (2013).

9 Zhao, H., Zheng, K., Li, Y. & Wang, J. A novel graph attention model for predicting frequencies of drug–side effects from multi-view data. *Briefings in Bioinformatics* **22**, bbab239 (2021).

10 Zhao, H. *et al.* A similarity-based deep learning approach for determining the frequencies of drug side effects. *Briefings in Bioinformatics* **23**, bbab449 (2022).
