# Supplementary material for: Identifying the serious clinical outcomes of adverse reactions to drugs by a multi-task deep learning framework
Source: Commun Biol. 2023 Aug 24;6:870. doi: 10.1038/s42003-023-05243-w (PMC10449791; doi:10.1038/s42003-023-05243-w)
Supplement: Supplementary file 2 — Description of Additional Supplementary Files [file 42003_2023_5243_MOESM2_ESM.pdf]

Description of Additional Supplementary Files

File name: Supplementary Data 1
Description: The known drug-ADR interactions in the benchmark dataset.

File name: Supplementary Data 2
Description: The seriousness classes of serious drug-ADR interactions in the benchmark dataset.

File name: Supplementary Data 3
Description: An independent test dataset from SIDER database.

File name: Supplementary Data 4
Description: An independent test dataset from OFFSIDES database.

File name: Supplementary Data 5
Description: The SMILES sequences of the drugs in the benchmark dataset.

File name: Supplementary Data 6
Description: The semantic descriptors of ADRs in the benchmark dataset.
