# Supplementary material for: Identifying the serious clinical outcomes of adverse reactions to drugs by a multi-task deep learning framework
Source: Commun Biol. 2023 Aug 24;6:870. doi: 10.1038/s42003-023-05243-w (PMC10449791; doi:10.1038/s42003-023-05243-w)
Supplement: Supplementary file 9 — Reporting Summary [file 42003_2023_5243_MOESM9_ESM.pdf]

Corresponding author(s): Jianxin Wang

Last updated by author(s): 7/20/2023

Reporting Summary

Nature Portfolio wishes to improve the reproducibility of the work that we publish. This form provides structure for consistency and transparency in reporting. For further information on Nature Portfolio policies, see our Editorial Policies and the Editorial Policy Checklist.

Statistics

For all statistical analyses, confirm that the following items are present in the figure legend, table legend, main text, or Methods section.

n/a	Confirmed	
☐	☒	The exact sample size (*n*) for each experimental group/condition, given as a discrete number and unit of measurement
☒	☐	A statement on whether measurements were taken from distinct samples or whether the same sample was measured repeatedly
☒	☐	The statistical test(s) used AND whether they are one- or two-sided<br>*Only common tests should be described solely by name; describe more complex techniques in the Methods section.*
☒	☐	A description of all covariates tested
☒	☐	A description of any assumptions or corrections, such as tests of normality and adjustment for multiple comparisons
☒	☐	A full description of the statistical parameters including central tendency (e.g. means) or other basic estimates (e.g. regression coefficient) AND variation (e.g. standard deviation) or associated estimates of uncertainty (e.g. confidence intervals)
☒	☐	For null hypothesis testing, the test statistic (e.g. *F*, *t*, *r*) with confidence intervals, effect sizes, degrees of freedom and *P* value noted<br>*Give P values as exact values whenever suitable.*
☒	☐	For Bayesian analysis, information on the choice of priors and Markov chain Monte Carlo settings
☒	☐	For hierarchical and complex designs, identification of the appropriate level for tests and full reporting of outcomes
☐	☒	Estimates of effect sizes (e.g. Cohen's *d*, Pearson's *r*), indicating how they were calculated

Our web collection on statistics for biologists contains articles on many of the points above.

Software and code

Policy information about availability of computer code

Data collection	The benchmark dataset in the manuscript are collected from two sources, i.e., known drug-ADR interactions from ADReCS database version v3.1 (http://bioinf.xmu.edu.cn/ADReCS/) and adverse event reports from united states. Food and drug administration Adverse Event Reporting System (FAERS, https://open.fda.gov/data/faers/).
Data analysis	This manuscripts utilize some open software and our scripts described in the Methods section. We use DGL (version 1.0), Python3, PyTorch (version 1.11.0).

For manuscripts utilizing custom algorithms or software that are central to the research but not yet described in published literature, software must be made available to editors and reviewers. We strongly encourage code deposition in a community repository (e.g. GitHub). See the Nature Portfolio guidelines for submitting code & software for further information.

Data

Policy information about availability of data

All manuscripts must include a data availability statement. This statement should provide the following information, where applicable:

- Accession codes, unique identifiers, or web links for publicly available datasets
- A description of any restrictions on data availability
- For clinical datasets or third party data, please ensure that the statement adheres to our policy

Data availability is as follows: All raw known drug-ADR interactions are collected from ADReCS database version v3.1 (http://bioinf.xmu.edu.cn/ADReCS/). All raw

adverse event reports are collected from the united states Food and drug administration Adverse Event Reporting System (FAERS, https://open.fda.gov/data/faers/).Drug-ADR interactions in the benchmark dataset (Supplementary Data 1), classes of serious clinical outcomes caused by drug-ADR interaction in the benchmark dataset (Supplementary Data 2), drug SMILES sequences (Supplementary Data 3), ADR semantic descriptions (Supplementary Data 4), and two independent test datasets (Supplementary Data 5 and 6). Any other relevant data are available from the authors upon reasonable request.

Research involving human participants, their data, or biological material

Policy information about studies with human participants or human data. See also policy information about sex, gender (identity/presentation), and sexual orientation and race, ethnicity and racism.

Reporting on sex and gender	N/A
Reporting on race, ethnicity, or other socially relevant groupings	N/A
Population characteristics	N/A
Recruitment	N/A
Ethics oversight	N/A

Note that full information on the approval of the study protocol must also be provided in the manuscript.

Field-specific reporting

Please select the one below that is the best fit for your research. If you are not sure, read the appropriate sections before making your selection.

☒ Life sciences ☐ Behavioural & social sciences ☐ Ecological, evolutionary & environmental sciences

For a reference copy of the document with all sections, see nature.com/documents/nr-reporting-summary-flat.pdf

Life sciences study design

All studies must disclose on these points even when the disclosure is negative.

Sample size	We collect all adverse event reports in FAERS from the third quarter of 2014 to the first quarter of 2022 and determined the seriousness of known drug-ADR interactions based on the Proportional Reporting Rate (PRR) approach. In total, the benchmark dataset contains a total of 141,752 drug-ADR interactions, covering 1,073 drugs and 893 ADRs. Among these, 58,429 drug-ADR interactions can cause serious clinical outcomes and have corresponding seriousness class labels.
Data exclusions	We extract the known associations between all drugs and Preferred Terms (PTs) of ADRs. We collect the primary suspect drug, ADRs, and its corresponding treatment outcome in each adverse event report. We count the number of drugs associated with ADRs in ADReCS and select ADRs with >50 associated drugs to maintain the high quality of the constructed dataset.
Replication	To evaluate the performance of our developed model, we utilize 10 times repeated 10-Fold Cross-Validation (10 × 10-Fold CV) and independent tests based on the benchmark dataset and two independent test sets, respectively (see Methods).
Randomization	Sample randomization was not relevant to this study. The machine learning training followed standard practices for separating training and testing datasets.
Blinding	Blinding was not relevant to this study, as all data is put together. The machine learning training followed standard practices for separating training and testing datasets.

Reporting for specific materials, systems and methods

We require information from authors about some types of materials, experimental systems and methods used in many studies. Here, indicate whether each material, system or method listed is relevant to your study. If you are not sure if a list item applies to your research, read the appropriate section before selecting a response.

Materials & experimental systems

n/a	Involved in the study
☒	☐ Antibodies
☒	☐ Eukaryotic cell lines
☒	☐ Palaeontology and archaeology
☒	☐ Animals and other organisms
☒	☐ Clinical data
☒	☐ Dual use research of concern
☒	☐ Plants

Methods

n/a	Involved in the study
☒	☐ ChIP-seq
☒	☐ Flow cytometry
☒	☐ MRI-based neuroimaging
